# Efficacy of ivermectin mass-drug administration to control scabies in asylum seekers in the Netherlands: A retrospective cohort study between January 2014 – March 2016

**DOI:** 10.1371/journal.pntd.0006401

**Published:** 2018-05-17

**Authors:** Dorien T. Beeres, Sofanne J. Ravensbergen, Annelies Heidema, Darren Cornish, Machiel Vonk, Leonie D. Wijnholds, Jessica J. H. Hendriks, Johanneke Kleinnijenhuis, Till F. Omansen, Ymkje Stienstra

**Affiliations:** 1 University of Groningen, Department of Internal Medicine, Infectious Disease Unit, University Medical Centre Groningen, Groningen, The Netherlands; 2 Primary Health Care Centre for Asylum Seekers (GCA), Ter Apel, The Netherlands; 3 Babylon Primary Health Care Services, Elst, The Netherlands; 4 Municipal health Care Centre Groningen, Groningen, The Netherlands; 5 Mediq pharmacy Ter Apel, Ter Apel, The Netherlands; 6 Health Care Service for Asylum seekers (Gezondheidscentrum Asielzoekers), Wageningen, The Netherlands; University of Tennessee, UNITED STATES

## Abstract

Scabies is a skin infestation with the mite *Sarcoptes scabiei* causing itch and rash and is a major risk factor for bacterial skin infections and severe complications. Here, we evaluated the treatment outcome of 2866 asylum seekers who received (preventive) scabies treatment before and during a scabies intervention programme (SIP) in the main reception centre in the Netherlands between January 2014 and March 2016. A SIP was introduced in the main national reception centre based on frequent observations of scabies and its complications amongst Eritrean and Ethiopian asylum seekers in the Netherlands. On arrival, all asylum seekers from Eritrea or Ethiopia were checked for clinical scabies signs and received ivermectin/permethrin either as prevention or treatment. A retrospective cohort study was conducted to compare the reinfestations and complications of scabies in asylum seekers who entered the Netherlands before and during the intervention and who received ivermectin/permethrin. In total, 2866 asylum seekers received treatment during the study period (January 2014 –March 2016) of which 1359 (47.4%) had clinical signs of scabies. During the programme, most of the asylum seekers with scabies were already diagnosed on arrival as part of the SIP screening (580 (64.7%) of the 897). Asylum seekers with more than one scabies episode reduced from 42.0% (194/462) before the programme to 27.2% (243/897) during the programme (RR = 0.64, 95% CI = 0.55–0.75). Development of scabies complications later in the asylum procedure reduced from 12.3% (57/462) to 4.6% (41/897). A scabies prevention and treatment programme at start of the asylum procedure was feasible and effective in the Netherlands; patients were diagnosed early and risk of reinfestations and complications reduced. To achieve a further decrease of scabies, implementation of the programme in multiple asylum centres may be needed.

## Author summary

The high number of asylum seekers arriving in Europe between 2014 and 2016 emphasized the importance to expand knowledge on best practice regarding health care provision and control of infectious diseases that may come with migration. Adequate control of scabies is important to prevent severe forms of scabies and prevent spread of the disease. Studies on interventions preventing or treating scabies in asylum seekers are very limited. To the best of our knowledge, this is the first study that provides data on an intervention targeting scabies in asylum seekers. This study shows that a scabies prevention and treatment programme at start of the asylum procedure is feasible and effective in the Netherlands. Patients were diagnosed early and risk of reinfestations and complications reduced. The programme may be used both to improve clinical practice and could serve as model for future policies on similar programmes.

## Introduction

In 2015, several outbreaks of scabies amongst asylum seekers at refugee camps and reception centres in Europe were reported [1–3]. Scabies is an infestation of the skin with the mite *Sarcoptes scabiei*. The mites are transmitted by close and prolonged skin to skin contact. Individuals with a severe form of scabies, such as crusted scabies, may harbour millions of mites and are highly contagious [4]. A manifestation of scabies mostly results in skin burrows, erythematous papules and generalized itching [5]. Secondary infections can lead to life-threatening complications including sepsis [6]. In 2017, the WHO recognized scabies as a neglected tropical disease [7]. Crowded conditions and frequently poor access to health care are major risk factors for scabies outbreaks and most likely gave rise to its recent emergence and spread in refugee camps [8].

Individual patients with uncomplicated scabies can be efficiently treated with permethrin or ivermectin [9]. However, mass outbreaks can be difficult to control and current knowledge on adequate prevention and treatment strategies for scabies in asylum seekers is limited. On a population level, mass drug administration with ivermectin was proven to be efficacious for the control of scabies in a highly endemic population in Fiji [10]. The greatest decline in the number of patients with scabies was seen in the treatment arm with 200ug/kg ivermectin [11]. Mass treatment with ivermectin and intensive active case finding results in long term control of scabies in community settings, but previous studies have not dealt with outbreaks as seen in asylum seekers [11,12].

In the Netherlands, the treating nurses and physicians noticed that asylum seekers originating from Eritrea and Ethiopia were particularly affected by scabies and frequently presented complications related to scabies. For this reason, an intervention programme for scabies was introduced in the national reception centres for asylum seekers in the Netherlands in July 2015.

This study aims to describe the epidemiology of scabies among asylum seekers that arrive in the Netherlands and to evaluate the effectiveness of a scabies intervention programme (SIP). We hypothesize that preventive treatment and early detection of scabies in a high-risk group reduces the number of episodes and the complication rate. Our data may conceivably help to improve the care of asylum seekers with scabies and may be useful to implement screening strategies in other vulnerable populations.

## Methods

### Ethics statement

This retrospective cohort study was evaluated by the ethics committee and was waived in accordance with Dutch legislation owing to its retrospective nature (University Medical Centre Groningen, METc number 2015/573). No written informed consent was obtained from patients for the use of retrospective data but patient information was anonymized and de-identified prior to analysis.

### Asylum seeking procedure in the Netherlands

The number of asylum seekers applications in the Netherlands was 29.790 in 2014, 56.370 in 2015, and 35100 in 2016 [13]. Among them, 13% (4056/29790) originated from Eritrea and Ethiopia in 2014, 15.7% (8682/58880) in 2015, and 9.2% (3235/35100) in 2016 [13]. The Netherlands operates a centralised system for asylum applications. Asylum seekers start their asylum procedure at one of the three reception centres in the Netherlands; Ter Apel, Budel and Veenhuizen. The majority of asylum seekers present at the reception centre in Ter Apel. Each asylum seeker centre in the Netherlands has its own primary health care centre organised by the national health care service for asylum seekers (GCA).

Within the first three days following arrival, individuals are identified, registered and then screened for active pulmonary tuberculosis. The regional public health services and the primary health care centre of asylum seekers in Ter Apel started an entrant screening programme, called the *‘Scabies Intervention Programme’* in July 2015 based on their observations of complicated scabies amongst asylum seekers arriving from Ethiopia and Eritrea.

### The scabies intervention programme

The SIP targeted asylum seekers from two countries. Asylum seekers from Eritrea and Ethiopia were selected based on the high prevalence of scabies observed among asylum seekers from these countries. Only the asylum seekers from these two countries were actively screened and were treated or given ivermectin/permethrin preventive treatment. It was practically not feasible to screen and treat all asylum seekers independent of their country of origin. In the first two days after arrival all asylum seekers from Eritrea and Ethiopia received information about scabies and its treatment with the help of a video in Tigrinya. Next their clothes were washed, they received temporary clothes, and were examined by a nurse for skin lesions. The nurses involved in the SIP worked at the health care centre which is based at the asylum centre. They gained experience during the earlier scabies outbreaks. This experience was complemented by training by the attending general practitioner based on a protocol developed by the National Institute of Public Health and the Environment [14]. The protocol advices on standard examination of the limbs. If skin abnormalities were observed on these body parts, a full body examination was performed if approved by the asylum seeker. Hand lenses were available, but not frequently used. During the intervention, the nurses would consult the attending physician if in doubt and the physician could refer the patient to a dermatologist if needed.

Asylum seekers with skin lesions or complaints compatible with scabies were treated with ivermectin at diagnosis and were invited to return to the health centre two weeks later in order to receive a second dose of ivermectin. Asylum seekers with complicated scabies or other skin diseases were referred to the general practitioner at the primary health care centre Ter Apel for immediate medical evaluation. All asylum seekers without a contraindication received ivermectin (0.2mg/kg, p.o.) under direct supervision of the nurse during the same visit. If ivermectin was contraindicated, for example in pregnant women and in children under the age of one, topical treatment with permethrin was given. All asylum seekers from Eritrea or Ethiopia without clinical signs and symptoms of scabies received a single dose of ivermectin. They travelled and arrived together and were considered to be contacts of the asylum seekers having scabies.

### Selection of participants

A retrospective cohort study was conducted at the primary health care centre located in the national reception centre for asylum seekers. The pharmacy records from the main reception center Ter Apel were used to identify all asylum seekers who received ivermectin/permethrin to treat or prevent scabies based on contact tracing between “January 1^st^ 2014 and March 14^th^ 2016. The medical records of these asylum seekers were followed over time (15–36 months) to check for alternative diagnoses and repeated use of ivermectin/permethrin. Medical records of asylum seekers are electronical patient files and can be accessed by health care workers in any asylum centre in the Netherlands after transfer of the asylum seeker. Data from asylum seekers were excluded from analysis if the pharmacy records in Ter Apel indicated use of ivermectin/permethrin without any mentioning of these medications or the diagnosis scabies in the medical file. The extraction of the ivermectin/permethrin prescriptions from the pharmacy record was independent of the country of origin of the asylum seeker. This data collection remained the same before and after introduction of the SIP.

Data on demographic characteristics; comorbidities; clinical scabies manifestations including complications; lab results (e.g. culture results from secondary infected lesions), and treatment for scabies as given in the intervention programme and the standard care (ivermectin, permethrin or both) have been extracted from medical records.

Scabies was defined as complicated if a patient had to be referred to the hospital, was treated for secondary infections, needed wound care, or received antibiotics for scabies complications. Rash and itch after scabies treatment may persist after successful treatment [15]. Complaints or symptoms of scabies within two weeks of treatment were therefore counted as part of the same episode.

### Statistical analysis

IBM SPSS Statistics for Windows, Version 23.0 was used to collect and analyse the data. Descriptive statistics were used for characteristics of all patients who visited the health care centre and registered as having scabies. A Relative Risk (CI) was calculated for the reinfestations before and during the SIP. The number of days until first scabies manifestation before and after the SIP was compared and calculated by Mann-Whitney-U.

## Results

### Study population and general characteristics

**Fig 1:**
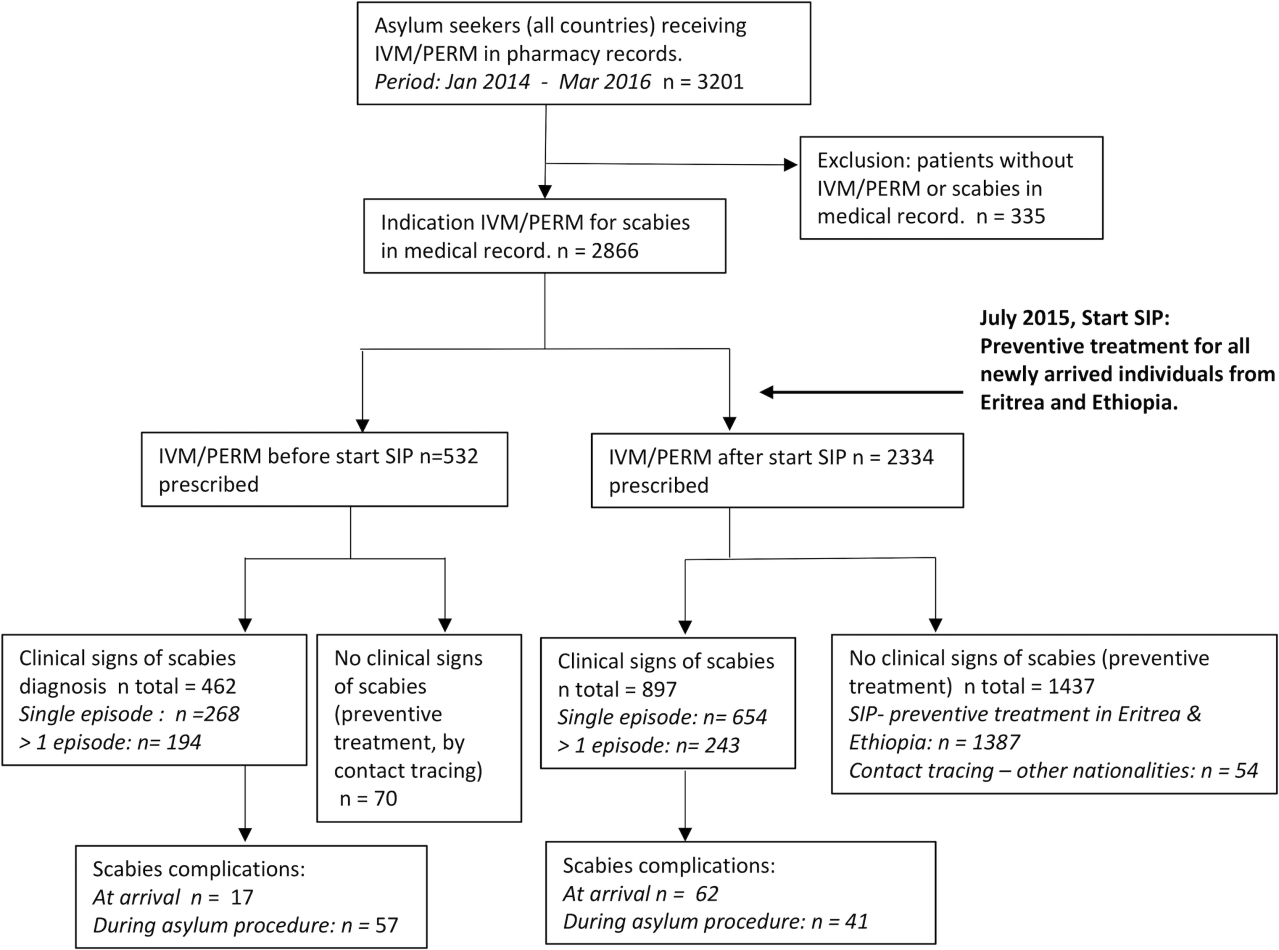
Flow chart of study participants January 2014 –March 2016, *IVM/PERM = ivermectin/permethrin.

During the study period, 3201 asylum seekers received ivermectin/permethrin at least once. Out of these asylum seekers, 2866 (89.5%) had a medical record with sufficient information to be included for further analysis (Fig 1). Asylum seekers who needed scabies treatment or prophylaxis before introduction of the SIP mainly originated from Ethiopia and Eritrea. The majority was male, and the mean (sd) age was 24 (± 9) years. Comorbidities were reported among 32 asylum seekers before SIP, and among 49 asylum seekers after the SIP. Comorbidities included tuberculosis (n = 47), hepatitis B/C (n = 14), HIV (n = 14), and diabetes (n = 7). There was no difference in the distribution of comorbidities before and after the SIP.

The indication for the use of ivermectin/permethrin in 2866 asylum seekers is summarized in Table 1. During the SIP, 580 (64.7%) of the 897 asylum seekers with signs and symptoms of scabies were identified through the scabies programme on arrival in the Netherlands and received treatment at the reception centre on the first day of arrival. The other 317 (35.3%) asylum seekers received their first treatment at one of the asylum health centres across the Netherlands after a median (IQR) of 83.5 days (46.8–178.8) after arrival.

**Table 1:** Indication for treatment in the study population before and after start of the SIP by gender and country of origin (total n = 2866).

		*Preventive treatment only (SIP/contact tracing^1^)*	*Single treatment for clinical scabies signs/symptoms*	*> 1 episodes with clinical signs/symptoms scabies*	*Developed scabies despite earlier preventive treatment^2^*	*Total*
***Before SIP (n = 532)***						
*Male (%)*		61.4	76.6	90.7	83.3	79.9
*Country of origin (n)*	Eritrea	55	137	136	13	341
	Ethiopia	13	95	44	4	156
	Other Africa	0	14	2	1	17
	Other; Eastern Europe & South America	0	0	1	0	1
	Other; Middle East & South Asia.	2	9	5	0	16
	Missing	0	1	0	0	1
	**TOTAL**	**70**	**256**	**188**	**18**^**2**^	**531**
***After initiation of the SIP (n = 2334)***						
*Male (%)*		54.3	75.9	81.1	65.8	62.3
*Country of origin (n)*	Eritrea	644	183	67	128	1022
	Ethiopia	745	260	91	135	1231
	Other Africa	33	8	4	4	49
	Other; Eastern Europe & South America	5	1	1	1	8
	Other; Middle East & South Asia	10	9	4	1	24
	**TOTAL**	**1437**	**461**	**167**	**269**^**2**^	**2334**

^1^Before the start of the SIP all preventive treatments were based on contact tracing. During the SIP contact tracing continued as part of standard care. All individuals from Eritrea and Ethiopia without signs or symptoms of scabies received preventive treatment on arrival.

^2^Respectively 6 and 76 before and during SIP developed multiple scabies episodes despite preventive treatment. These patients are counted in this column only.

### Clinical manifestations and diagnosis of scabies

**Fig 2:**
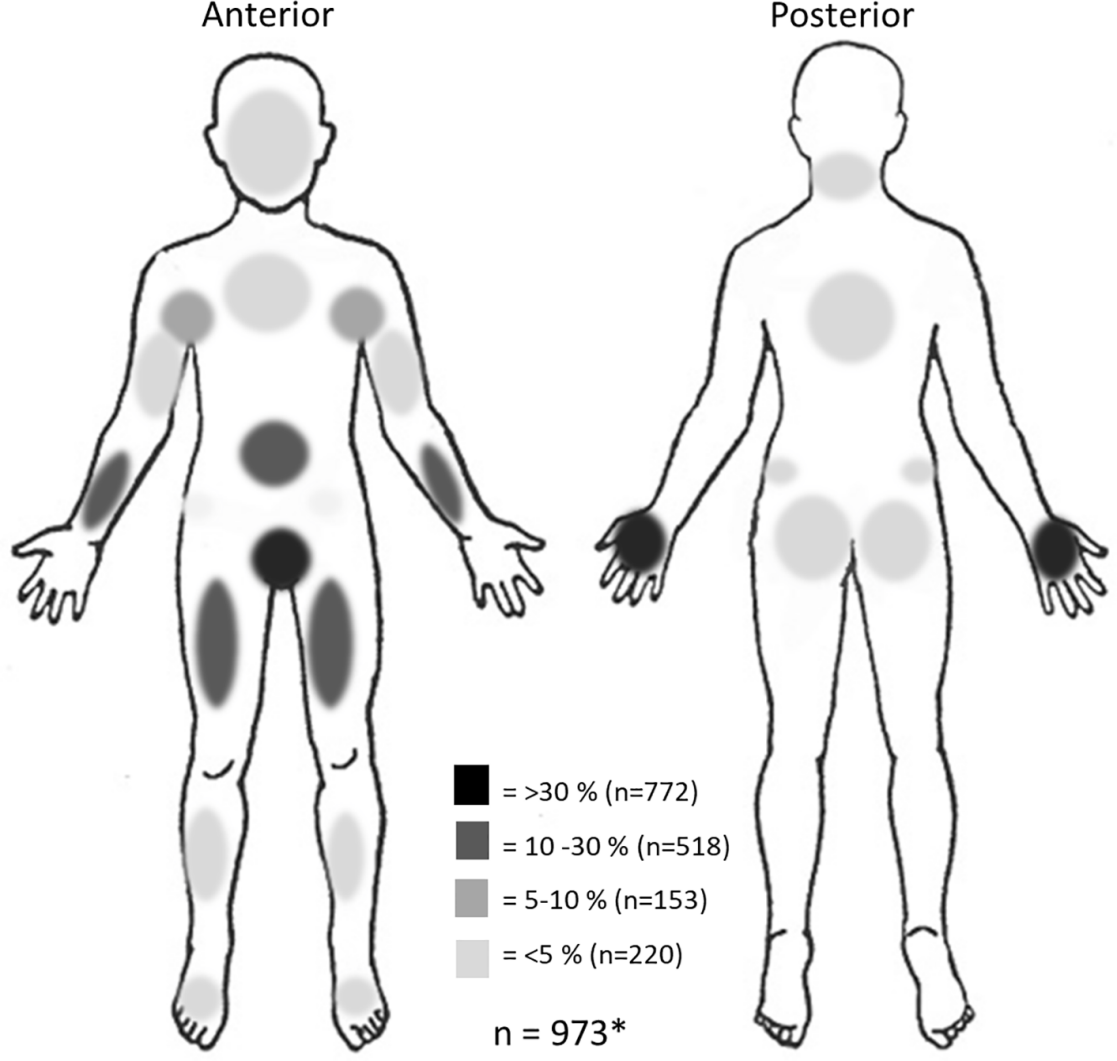
Density map of the distribution of scabies signs and symptoms at different body parts. *locations of scabies signs were missing in medical record for 386 patients.

An overview of the clinical manifestations at the first episode of the 1359 scabies patients is given in Table 2. 33.5% of the asylum seekers presented with more than one sign or symptom of scabies. Itch was reported in 77% of the episodes. Other signs or symptoms include burrows and other visible skin defects such as excoriations. Episodes with atypical presentations of scabies in the face (n = 17) and neck (n = 13) were noticed in combination with lesions on the hand and in the genital area (Fig 2).

**Table 2:** Clinical presentation of scabies and complications before and after start of the programme.

	*Before SIP* *n = 462*	*During SIP* *n = 897*	*Total* *n = 1359*
***Clinical presentation***	*n (%)*	*n (%)*	*n (%)*
*Itch*	371 (80.3)	679 (75.7)	1050 (77.3)
*Burrows*	49 (10.6)	78 (8.7)	127 (9.3)
*Other visible skin defects*	161 (34.8)	282 (31.4)	443 (32.6)
*Secondary infections*	62 (13.4)	87 (9.7)	149 (11.0)
*Crusted scabies*	3 (0.006)	8 (0.009)	11 (0.009)
*Missing in medical record*	190	377	567
***Complications related to scabies***			
*Reported or diagnosed on arrival*	17 (3.7)	62 (6.9)	79 (5.8)
*Reported during asylum procedure*	57 (12.3)	41 (4.6)	98 (7.2)

Of the 462 asylum seekers with signs and symptoms of scabies before start of the SIP, 12.3% presented with complications of scabies during their asylum procedure, compared to 4.6% of the 897 asylum seekers with signs of scabies after the start of the SIP (RR = 0.37, 95% CI = 0.25–0.54). Asylum seekers with complications presented after a median (IQR) of 6.0 (1–28) days after arrival before start of the SIP and 0 (0–32) days during the SIP (*U =* 2863.5, p *=* 0.005).

None of the asylum seekers with skin problems interpreted as being scabies, proved to have an alternative diagnosis during follow up. Before start of SIP, only 13 asylum seekers were initially misdiagnosed and were diagnosed with scabies at their second visit.

### Treatment

As described in the previous section, 1359 scabies patients received treatment for the clinical manifestations at the first disease episode. Ivermectin was given 1232 times and permethrin was given 105 times. Twenty-two patients (not during the SIP) received a combined treatment with ivermectin and permethrin due to the disease severity. The protocol included a second dose of treatment after two weeks for the asylum seekers that presented with clinical signs and symptoms of scabies. Among them, 34% (461/1359) had a notification of the intake of a second dose of treatment in their medical record. Scabies reinfestations were more common among individuals reporting the intake of a single dose of ivermectin 40.0% (286/717) compared to the individuals that report the intake of a second dose of ivermectin 27.3% (175/642) (RR = 1.37 95% CI (1.20–1.57).

Antibiotics were prescribed in 117 (68.5%) of the 149 asylum seekers with secondary infections. Most frequently prescribed antibiotics were flucloxacillin (55), amoxicillin/clavulanic acid (29), and fusidic acid (29). Incision and drainage of abscesses were performed in 35 patients. Wound care was needed in 85 patients. Cultures were performed in 12 asylum seekers with secondary infections at the health care centre of the national reception centre. Cultures showed Methicillin-Sensitive *Staphylococcus aureus* (MSSA) in 9 patients and Methicillin-Resistant *Staphylococcus aureus* (MRSA) in 3 patients.

### Reinfestations

Reinfestations were more common amongst scabies patients before introduction of SIP. During the SIP, 27.2% (243/897) of the scabies patients had more than one disease episode versus 44% (194/462) of the scabies patients before start of the SIP (RR = 0.64, 95% CI = 0.55–0.75).

In total 1691 asylum seekers received preventive treatment on arrival, of which 269 (15.9%) asylum seekers were diagnosed with scabies later in their asylum procedure (after a median (IQR) days of 75 (44–146)). The majority (242 of the 269 (90.0%)) reported during their stay in asylum health centres other than the national receptions centres Ter Apel and Budel. There was no proactive scabies programme in these asylum centres.

## Discussion

Frequent observations of scabies and its complications were reported amongst Eritrean and Ethiopian asylum seekers arriving in the Netherlands. Introduction of a scabies prevention and treatment programme on arrival was feasible and resulted in early detection of asylum seekers with scabies. Itch and burrows were the most common clinical manifestations. Atypical scabies presentations in the face and neck were also noticed. The number of scabies reinfestations after treatment and the number of complicated forms of scabies reduced after introduction of the programme. Asylum seekers who received ivermectin/permethrin on arrival but developed scabies in other asylum centres across the Netherlands, presented after a median of 2.5 months after arrival.

Introduction of the SIP was effective in the prevention of scabies outbreaks. Given the high number of the asylum applications in 2014 and 2015 [13] a target group for the intervention was selected. The few scabies manifestations amongst asylum seekers from countries other than Ethiopia and Eritrea suggests that the target group of the SIP was well chosen.

Reinfestations after scabies treatment were common before the programme started. After introduction, the number of scabies episodes per person reduced significantly. Prompt treatment of new cases is important for successful scabies control in institutional settings [16,17]. In other institutional settings, a public jail and a hospital ward, collective treatment of persons with oral ivermectin in both a treatment and prophylaxis setting appeared to be effective in the treatment of scabies [18,19]. In our study, newly developed scabies episodes were mainly seen in asylum health centres other than the national reception centres Ter Apel and Budel. These centres did not have a scabies prevention and treatment programme. This indicates that to achieve a further decrease of scabies, implementation of the programme in multiple asylum centres may be needed.

Definitive diagnosis of scabies relies on microscopic identification of the mites or eggs [20]. However, scabies diagnosis based on clinical recognition by well-trained nurses seemed efficient in this setting with high pre-test probability of having scabies if asylum seekers had itching or typical skin lesion; no scabies patients received alternative diagnoses during follow-up. Diagnostic accuracy of scabies diagnosis based on the combination of symptoms and signs recognition was also proven in a study performed in Mali and Senegal [21]. Over diagnosis of scabies during the SIP could be a factor that contributed to the reduced reinfestations since start of the SIP, but the data collected in this setting with a high pre-test probability of scabies suggests a good positive predictive value.

Secondary infections and complications are common amongst scabies patients [22]. Introduction of the programme reduced the number and the severity of the complicated forms of scabies. It has been suggested that scabies mites provide favourable conditions for onset of *S*. *aureus* co-infection [23]. Severe forms of scabies such as bacterial super infections and crusted scabies were reported in this study. Culture of the secondary infections tested positive for both the presence of MSSA and MRSA. However, the number of coinfections is relatively low compared to other studies that describe secondary infections related to scabies [24].

A limitation of this study is the unknown duration of stay of the asylum seekers in the asylum centers. Therefore, it is impossible to calculate the exact incidence rate of scabies. Due to the frequent relocations of asylum seekers to other asylum centers within the Netherlands without a proactive scabies programme, reinfestations may occur by contact with untreated contagious individuals. Scabies manifestations after preventive treatment occurred after a minimum time period of two and a half months, suggesting that initial preventive treatment was effective. Other factors may have led to a reduction in the rate of reinfestations which are not included in this study, e.g. improved housing of asylum seekers. However, we are unaware of such changes in policy that could lead to a reduction in the rate of reinfestations.

Finally, participation in the SIP was on a voluntary basis. The number of patients that refused participation in the programme is unknown and these individuals could influence the efficacy of the programme. However, health care workers did not observe asylum seekers who refused participation which suggests that refusal rate was low.

Controlling scabies amongst asylum seekers is important to reduce the risk of complicated cases and to prevent the spread of scabies amongst asylum seekers at asylum centres in the Netherlands. The centralized health care system for asylum seekers that is used in the Netherlands allows accurate follow up of asylum seekers and provides unique retrospective data on the programme.

The systematic examination of the skin by nurses as part of this programme also results in early identification of other skin diseases such as chronic wounds, cutaneous leishmaniasis and eczema and leads to referral to the general practitioner in case of other systemic signs like fever. This programme therefore has the potential for an integrated control of skin conditions and human-to-human transmittable diseases, such as louse borne relapsing fever [25]. To our knowledge this is the first study to investigate the effect of a screening and mass-drug administration programme for scabies control in asylum seekers. The programme helped to be up to scratch with itchy outbreaks by early detection and reducing the number of reinfestations and complications.

## Supporting information

S1 Checklist STROBE checklist.
(DOCX)
